# Prognostic factors for low birthweight repetition in successive pregnancies: a cohort study

**DOI:** 10.1186/1471-2393-13-20

**Published:** 2013-01-23

**Authors:** Iândora Krolow Timm Sclowitz, Iná S Santos, Marlos Rodrigues Domingues, Alicia Matijasevich, Aluísio J D Barros

**Affiliations:** 1 Post-graduate Program in Epidemiology, Departamento de Medicina Social, Faculdade de Medicina, Federal University of Pelotas, Rua Marechal Deodoro, 1160 – 3rd floor, Pelotas, 96020-220, Brazil

**Keywords:** Prognostic factors, Low birthweight recurrence, Low birthweight, Preterm, Antenatal care

## Abstract

**Background:**

To identify prognostic factors associated with recurrence of low birthweight (LBW) in successive gestations, a study was carried out with a subsample of mothers enrolled in the 2004 Pelotas Birth Cohort.

**Methods:**

Data were collected by hospital-based interviews. Newborns were weighed and measured. Gestational age was defined according to the date of last menstrual period, ultra-sound scan before the 20^th^ week of pregnancy or the Dubowitz method. Mothers who reported at least one LBW newborn in the two previous gestations were included. Prevalence ratios (PR) and 95% confidence intervals were estimated from Poisson Regression. All estimates were adjusted for parity.

**Results:**

A total of 4558 births were identified in 2004, and 565 met inclusion criteria, out of which 86 (15.2%) repeated LBW in 2004. Among mothers with two LBW babies before 2004, 47.9% presented LBW recurrence. Belonging to the highest socio-economic stratum (PR 0.89; 0.01-0.46) and gaining ≥ 10 kg during pregnancy (PR 0.09; 0.01-0.77) were protective against LBW recurrence. Higher risk of LBW recurrence was observed among mothers with higher parity (≥3 previous deliveries; PR=1.93; 95% CI 1.23-3.02); who had given birth to a previous preterm baby (PR=4.01; 2.27-7.10); who delivered a female newborn in current gestation (PR=2.61; 1.45-4.69); and that had not received adequate antenatal care (PR=2.57; 1-37-4.81).

**Conclusion:**

Improved quality of antenatal care and adequate maternal weight gain during pregnancy may be feasible strategies to prevent LBW repetition in successive pregnancies.

## Background

Low birthweight (LBW) defined as birth weight lower than 2500 grams is an important infant mortality and morbidity predictor [1,2]. Birthweight derives basically from two processes: length of pregnancy and fetal growth rate. Hence, LBW might be caused either by a short gestational period (preterm birth) or by intrauterine growth restriction (small for gestational age - SGA) and even by a combination of both factors. A meta-analysis about LBW released in 1987 [3] based on English and French literature, identified 43 possible risk factors to the occurrence of LBW. The same study pointed out the fact that some mothers repeatedly deliver babies of similar birthweights and gestational ages, suggesting the existence of common underlying risk factors or genetic characteristics that play a role in the causation of such outcomes.

The identification of factors involved in the repetition of LBW is an attempt to understand the causal links that increase the chance of some women to be more susceptible to give birth to a LBW child. Previous LBW and preterm birth are usually investigated as risk markers in studies aiming to assess risk factors for LBW and preterm births. Studies planned to specifically explore LBW repetition or gestational age repetition are lacking in the literature. After a systematic review for publications in the last 40 years only six studies were identified [4]. The present study was planned to investigate prognostic factors for LBW repetition among mothers from the 2004 Pelotas Birth Cohort. Only mothers with a history of previous LBW newborn were eligible to the study.

## Methods

This study was carried out within a larger project – The 2004 Pelotas Birth Cohort [5]. Data were collected by the occasion of the perinatal interview. In 2004, five maternity hospitals, where all deliveries take place in the city of Pelotas, Brazil, were visited on a daily basis. During their stay at the hospital mothers responded to a standardized questionnaire about demographic, socioeconomic, reproductive, behavioral, and prenatal care characteristics, besides gestational and pre-gestational morbidities. Methods describing the 2004 Pelotas Birth Cohort in detail have been previously published [5,6].

Only mothers who reported at least one previous LBW newborn in the two immediate pregnancies before 2004 were included in the current analyses. The inclusion criteria also demanded that these pregnancies were not multiple and did not result in malformation or abortion. From the 2004 births, primiparas, malformation cases and multiple pregnancies were also excluded.

Newborns were weighed with digital pediatric scales soon after labor. Scales were checked for accuracy weekly by using standard weights. Length measurements were performed in supine position and thoracic, cephalic and abdominal circumferences were obtained with inelastic measuring tapes. Gestational age was calculated by an algorithm starting with the date of last menstrual period. When last menstrual period was missing or implausible, ultra-sound information was used as long as the exam had been done before the 20^th^ week of pregnancy. When ultra-sound was missing too, the Dubowitz score was used. Interviewers in charge of data collection were graduated nutritionists, previously trained for a period of seven days. During data collection interviewers were re-evaluated periodically to confirm quality of interview and of newborn physical examination. Interviewers were allocated in a way that each member of the team worked in every hospital, changing working location and shifts weekly. In order to assure data quality, after hospital discharge a fieldwork supervisor repeated 10% of the interviews by telephone, using a short version of the questionnaire. Around 5% of the mothers were contacted while still in the hospital to answer a similar short version of the questionnaire; the latter was done by a gynecologist (fieldwork supervisor) who also performed a Dubowitz examination.

Five social classes, labeled A (richest) to E (poorest) based on nine assets, presence of domestic employees and the education level of the household head were defined according to the Brazilian Research Companies Association ( http://www.abep.org/codigosguias/ABEP_CCEB.pdf). Information on mother’s and father’s schooling (accomplished years of formal education) and maternal marital status (mother currently living with or without a partner) was collected. Maternal biologic variables included age (at the moment of the interview), skin color (white, non-white or other - observed by the interviewer), height (in cm), LBW born-mothers (yes/no; according to the reported maternal own weight at birth < 2500 grams), and preterm born-mothers (yes/no; according to the reported maternal own gestational age at birth < 37 weeks of pregnancy). For the father, current age was collected. Reproductive and behavioral maternal characteristics gathered were parity (number of previous births), smoking (everyday during each trimester of the pregnancy, regardless of the number of cigarettes smoked), caffeine consumption (coffee ingestion for each trimester of pregnancy), abortion history (yes/no), interpartal interval (months between last and current delivery), newborn sex, preterm history (yes/no), antenatal care (classified as adequate, inadequate or intermediate according to Kessner Index, adapted by Takeda) [7]. Morbidities evaluation for the current pregnancy included mother’s arterial hypertension (yes/no), anemia (yes/no), miscarriage threat (yes/no), premature labor (yes/no), vaginal bleeding in the last trimester (yes/no), and urinary tract infection (yes/no). Weight gain during the pregnancy (from the antenatal mother’s card - in kg) was also collected.

**Figure 1:**
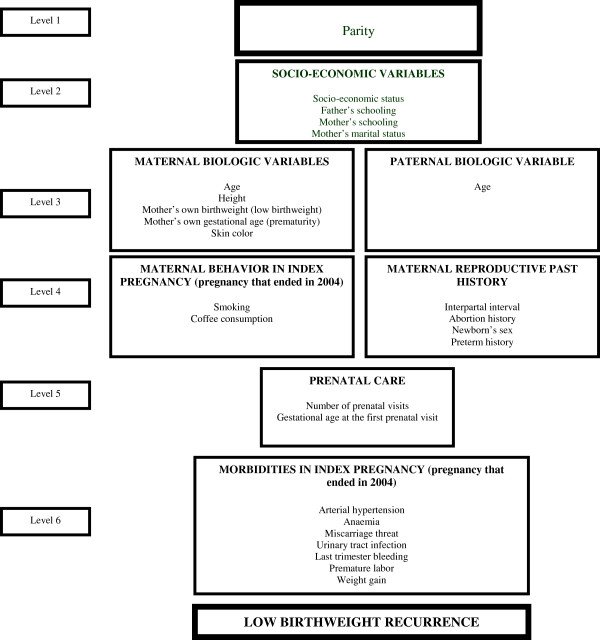
Hierarchical model of analysis.

Newborns with gestational age of less than 37 weeks were considered as preterm and babies weighing less than 2500 grams at birth were classified as LBW. Crude and multivariable analyses were carried out with Stata 11.0. Because the prevalence of repeated LBW was high, prevalence Ratios (PR) and 95% Confidence Intervals (95%CI) were estimated through robust Poisson Regression, to prevent overestimation of PR obtained through logistic regression [8]. For multivariable analysis, variables were entered in the model according to a six-level pre-defined hierarchical model (Figure 1). Level one was composed by the variable “parity”; level two included socio-economic characteristics; level three, maternal biological characteristics and fathers’ age; level four, maternal behavior, maternal reproductive variables and newborn’s sex; level five, prenatal care characteristics; and level six, pregnancy morbidities. Because parity is strongly associated to birth weight [9] and to increased “opportunity” to have a LBW baby before, all other variables were adjusted for parity (Figure 1). For each level, a backward regression was run and the significance level for maintenance in the model was set at 20% (p < 0.20). Variables from level two were adjusted for each other and for parity. Variables from level three were adjusted for each other, parity and for variables from level two that remained statistically significant. The same procedure was employed consecutively for variables from levels four to six. The outcome of interest was the delivery in 2004 of a LBW baby from a mother with history of at least one LBW newborn from a prior pregnancy. All other variables were explored as independent variables.

Study protocol was approved by the Medical Ethics Committee of the Federal University of Pelotas. Written consent was obtained from mothers before the interview.

## Results

A total of 4558 births were identified in Pelotas in 2004, from which, 565 were eligible according to the inclusion criteria (at least one LBW baby prior to 2004). From the 565 mothers, 86 (15.2%) gave birth to a LBW baby in 2004 and therefore were considered positive for the outcome. Among mothers with two LBW births prior to 2004, recurrence of LBW was 47.9%.

Table 1 presents a sample description according to parents’ socio-economic and biological variables. Around 57% of the studied women belonged to D and E economic classes. LBW recurrence was more commonly observed among E class members (22.2%), the poorest mothers. Regarding schooling, 23% of the parents attended up to four years of formal education and 88.5% of the women were living with a partner. Recurrence of LBW was higher among newborns from parents with lower education level.

**Table 1:** Low birthweight recurrence [LBWR]* according to maternal socio-economic and biological characteristics

**Variable**	**N [%]**	**LBWR [%]**	**p**
**Economic status**^**a**^**[n = 408]**^**b**^			0.008 ^c^
A and B	58 [14.2]	2 [3.5]	
C	119 [29.2]	9 [7.5]	
D	168 [41.2]	26 [15.5]	
E (poorest)	63 [15.4]	14 [22.2]	
**Father’s schooling (years) [n = 481]**^**b**^			0.04 ^c^
0 – 4	113 [23.5]	23 [20.3]	
5 – 8	208 [43.2]	31 [14.9]	
≥ 9	160 [33.3]	18 [11.2]	
**Mother’s schooling (years) [n = 563]**^**b**^			< 0.001 ^c^
0 – 4	132 [23.4]	32 [24.2]	
5 – 8	261 [46.4]	39 [14.9]	
≥ 9	170 [30.2]	15 [8.8]	
**Marital status [n = 565]**			0.74 ^d^
Living with partner	65 [11.5]	9 [13.8]	
Living without partner	500 [88.5]	77 [15.4]	
**Mother’s age [n = 564]**^**b**^			0.71 ^c^
15 - 20	82 [14.5]	13 [15.8]	
21 - 25	162 [28.7]	27 [16.7]	
26 - 30	136 [24.1]	17 [12.5]	
31 - 35	108 [19.1]	17 [15.7]	
36 - 44	76 [13.5]	11 [14.5]	
**Mother’s skin color [n = 565]**			0.65 ^d^
White	384 [68.0]	61 [15.9]	
Non-white	142 [25.1]	21 [14.8]	
Other	39 [6.9]	4 [10.3]	
**Father’s age [n = 552]**^**b**^			0.53 ^c^
16 - 20	23 [4.2]	6 [26.1]	
21 - 25	123 [22.3]	19 [15.4]	
26 - 30	127 [23.0]	17 [13.4]	
31 - 35	134 [24.3]	19 [14.2]	
36 - 64	145 [26.8]	22 [15.2]	
**Mother’s height [cm] [n = 475]**^**b**^			0.63^c^
<150	23 [4.8]	6 [26.1]	
150 - 154	60 [12.6]	10 [16.7]	
155 - 159	88 [18.5]	15 [17.0]	
160 - 164	95 [20.0]	10 [10.5]	
≥165	209 [44.0]	37 [17.7]	
**LBW born-mother [n = 377]**^**b**^			0.004 ^d^
No	320 [84.9]	39 [12.2]	
Yes	57 [15.1]	15 [26.3]	
**Preterm born-mother [n = 510]**^**b**^			0.004^d^
No	473 [92.7]	63 [13.3]	
Yes	37 [7.3]	11 [29.7]	

* LBWR defined as birth weight < 2,500 g in the index pregnancy (pregnancy that ended in 2004) amongst women with at least one previous LBW newborn in the two immediate pregnancies before the pregnancy that ended in 2004.

a: Brazilian Research Companies Association; b: Indicates missing values; c: Linear trend; d: Chi-squared test for heterogeneity.

Regarding maternal own birthweight, 15.1% of the women were LBW born-mothers, among whom recurrence of LBW in 2004 was 26.3% in comparison to 12.2% among non-LBW born-mothers. Seven percent of the women were preterm born-mothers and the recurrence of LBW among them was 29.7% against 13.3% among non-preterm born-mothers.

Table 2 shows reproductive and behavioral maternal characteristics. More than 50% of the women gave birth only once previously to 2004. Among them, 11.5% presented LBW recurrence. Frequency of LBW increased with increased parity. Regarding maternal behavior, 32% smoked in the last trimester of current pregnancy. Recurrence of LBW was higher among smokers (21.3%) than among non-smokers (12.3%). More than one third of the mothers reported a previous preterm birth. Recurrence of LBW was more than 3 times higher among them than among mothers with no history of previous preterm birth. Antenatal care was inadequate or intermediate for 43.4% of the mothers. LBW repetition was higher for them (21.6%) than for those who had an adequate care (10.3%).

**Table 2:** Low birthweight recurrence [LBWR]* according to parity, maternal reproductive past history, maternal behaviors, and prenatal care in the index pregnancy

**Variable**	**N [%]**	**LBWR [%]**	**p**
**Parity [previous births] [n = 565]**			<0.001 ^a^
1	322 [57.0]	37 [11.5]	
2	117 [20.7]	21 [17.9]	
≥3	126 [22.3]	28 [22.2]	
**Smoking in the 3**^**rd**^**trimester [n = 565]**			
No	382 [67.6]	47 [12.3]	<0.001 ^b^
Yes	183 [32.4]	39 [21.3]	
**Coffee consumption in the 3**^**rd**^**trimester [n = 565]**			0.53 ^b^
No	181 [32.0]	30 [16.6]	
Yes	384 [68.0]	56 [14.6]	
**Abortion history [n = 565]**			0.3 ^b^
No	437 [77.3]	63 [14.4]	
Yes	128 [22.7]	23 [18.0]	
**Interpartum interval [n = 544]**^**c**^			0.28 ^b^
< 24 months	117 [21.5]	21 [17.9]	
≥ 24 months	427 [78.5]	60 [14.0]	
**Preterm birth in previous pregnancy [n = 532]**^**c**^			<0.001 ^b^
No	345 [64.8]	28 [8.1]	
Yes	187 [35.2]	54 [28.9]	
**Prenatal care [n = 565]**			<0.001 ^b^
Adequate	320 [56.6]	33 [10.3]	
Intermediate or Inadequate	245 [43.4]	53 [21.6]	

* LBWR defined as birth weight < 2,500 g in the index pregnancy (pregnancy that ended in 2004) amongst women with at least one previous LBW newborn in the two immediate pregnancies before the pregnancy that ended in 2004.

a: linear trend; b: chi squared test for heterogeneity; c: indicates missing values.

Table 3 describes maternal gestational morbidities, preterm labor, gestational weight gain, and newborn’s sex in the index pregnancy. LBW repetition was more frequent among newborns from mothers who reported anemia (20.1% against 12.8%), urinary tract infection (19.6% versus 12.0%), and weight gain during pregnancy lower than 7 kg in comparison to those who gained 10 kg or more (25.4% versus 2.1%). Recurrence of LBW was higher for female newborns.

**Table 3:** Low birthweight recurrence [LBWR]* according to pregnancy morbidities, preterm labor, gestational weight gain, and newborns’ sex

**Variable**	**N [%]**	**LBWR [%]**	**p**
**Arterial hypertension [n = 562]**^**a**^			0.74 ^b^
No	410 [72.9]	64 [15.6]	
Yes	152 [27.1]	22 [14.5]	
**Anemia [n = 555]**^**a**^			0.02 ^b^
No	179 [32.5]	36 [20.1]	
Yes	376 [67.7]	48 [12.8]	
**Miscarriage threat [n = 564]**^**a**^			0.96 ^b^
No	491 [87.1]	75 [15.3]	
Yes	73 [12.9]	11 [15.1]	
**Preterm labor [n = 565]**			0.01 ^b^
No	422 [74.7]	55 [13.0]	
Yes	143 [25.3]	31 [21.7]	
**Last trimester vaginal bleeding [n = 565]**			0.82 ^b^
No	516 [91.3]	78 [15.1]	
Yes	49 [8.7]	8 [16.3]	
**Urinary tract infection [n = 561]**^**a**^			0.01^b^
No	332 [59.2]	40 [12.0]	
Yes	229 [40.8]	45 [19.6]	
**Gestational weight gain [n = 403]**^**a**^			<0.001^c^
< 7 kg	236 [49.6]	60 [25.4]	
7 – 9.9 kg	99 [20.8]	12 [12.1]	
≥ 10 kg	141 [29.6]	3 [2.1]	
**Newborn’s sex [n = 563]**^**a**^			0.001^b^
Male	286 [50.8]	29 [10.1]	
Female	277 [49.2]	56 [22.2]	

* LBWR defined as birth weight < 2,500 g in the index pregnancy (pregnancy that ended in 2004) amongst women with at least one previous LBW newborn in the two immediate pregnancies before the pregnancy that ended in 2004.

a: indicates missing values; b: chi squared test for heterogeneity; c: linear trend.

Table 4 presents crude and adjusted PR with 95%CI. The first level of the analysis included parity alone. PR for LBW recurrence was almost twice as high (1.93; 95%CI 1.23-3.02) among women with three or more gestations in comparison to women with only one gestation prior to 2004. In the second level, LBW recurrence was associated to economic status, being higher in the poorest class. None of the variables in the third level (maternal biologic variables and father’s age) was statistically associated to recurrence of LBW. In the fourth level, LBW recurrence was associated to maternal preterm birth history in past pregnancies in the crude and adjusted analysis. Mothers with preterm birth history had a fourfold increase in probability to repeat LBW in the current pregnancy. Compared to male newborns, female presented a 2.61 fold increase in risk of being LBW.

**Table 4:** Crude and adjusted prevalence ratios (PR) with 95% confidence intervals analyses for low birthweight recurrence [LBWR]*

**Variable**	**Crude PR**^**a **^**[CI95%]**	**Adjusted PR**^**a **^**[CI95%]**	**p**
**Parity [previous births] (level 1)**			0.01
1	1.0 Reference		
2	1.56 [0.95,2.55]		
≥3	1.93 [1.23,3.02]		
**Economic status (level 2)**			0.045
A and B	0.15 [0.03,0.65]	0.89 [0.01,0.46]	
C	0.34 [0.15,0.74]	0.25 [0.10,0.60]	
D	0.69 [0.38,1.24]	0.60 [0.32,1.10]	
E (poorest)	1.0 Reference	1.0 Reference	
**Father’s schooling (years) (level 2)**			0.13
0 – 4	1.80 [1.02,3.19]	0.54 [0.25,1.15]	
5 – 8	1.32 [0.76,2.28]	0.47 [0.22,1.00]	
≥ 9	1.0 Reference	1.0 Reference	
**Preterm birth history (level 4)**			<0.001
No	1.0 Reference	1.0 Reference	
Yes	3.55 [2.33,5.41]	4.01 [2.27,7.10]	
**Newborn’s sex (level 4)**			<0.001
Male	1.0 Reference	1.0 Reference	
Female	1.99 [1.31,3.02]	2.61 [1.45,4.69]	
**Prenatal care (level 5)**			0.003
Adequate	1.0 Reference	1.0 Reference	
Intermediate or Inadequate	2.09 [1.40,3.13]	2.57 [1.37,4.81]	
**Miscarriage threat (level 6)**			0.14
No	1.01 [0.56,1.81]	2.00 [0.77,5.14]	
Yes	1.0 Reference	1.0 Reference	
**Gestational weight gain (kg) (level 6)**			0.04
< 7	1.0 Reference	1.0 Reference	
7 – 9.9	1.47 [0.26,0.84]	0.59 [0.31,1.13]	
≥ 10	0.83 [0.02,0.26]	0.09 [0.01,0.77]	

* LBWR defined as birth weight < 2,500 g in the index pregnancy (pregnancy that ended in 2004) amongst women with at least one previous LBW newborn in the two immediate pregnancies before the pregnancy that ended in 2004.

a: Prevalence Ratios; *CI*: Confidence Interval.

In the fifth level, women with intermediate or inadequate quality of antenatal care presented a PR = 2.57 and in the sixth level, mothers with weight gain of 10 kg or more had a probability of LBW recurrence almost 100% smaller than that of women gaining less than 7 kg during pregnancy.

## Discussion

Frequency of LBW in the group of mothers with at least one previous pregnancy ending in a LBW newborn (15.2%) was 1.5 times higher than the observed for the entire 2004 Birth Cohort population (10.0%) [5], highlighting the greater risk for LBW within this group. LBW recurrence was associated to higher maternal parity, lower socio-economic class, maternal history of prior preterm birth, female sex of the newborn, low quality of antenatal care, and low maternal weight gain during pregnancy.

It’s difficult to establish comparisons between this study and findings from other authors due to different methodologies and different outcome definitions. Findings from other studies showed that LBW recurrence was associated to mother’s smoking [10], shorter interpartal interval [10,11], black skin color [10], and mother’s ages older than 30 [10]; whereas maternal body weight higher than on previous pregnancy [10], and children from the same father but from different mothers were protective factors for recurrence of LBW [12]. LBW recurrence was not influenced by changing socio-economic status, residence (rural/urban) or father’s occupation [12].

Others have found that recurrence of SGA infants in successive gestations was associated to maternal ages younger than 20 and older than 35 [13], lower maternal education level [13], a non-qualified father’s occupation [13], arterial hypertension [14] and mother’s drug addiction [14], whereas studies that analyzed preterm birth repetition reported association with the recurrence of risk factors such as premature rupture of membranae, chorioamnionitis and pre-eclampsia [15]. In another study, association was found with premature labor and interpartal interval shorter than twelve months [11].

Most of these studies however compared mothers with repeated outcome (LBW, preterm birth or SGA newborns) against mothers who had given birth only to normal weighed and term infants, thus exploring risk factors for LBW occurrence, not for LBW recurrence. Nonetheless, risk factors for LBW recurrence may not necessarily be the same as prognostic factors for repetition of LBW among mothers who have already experienced the delivery of a LBW newborn. Because LBW is a recognized risk marker for subsequent LBW delivery, identification of modifiable prognostic factors among this high risk group of mothers is important for recurrence prevention. The historical cohort design of our study analyzing a group of women with previous experience of a LBW delivery is appropriate to uncover prognostic factors for repeated LBW in a subsequent pregnancy.

With regard to the association with low socio-economic class, Bakketeig et al. [13] similarly have shown the same association with SGA. Conversely, a Danish study failed to prove this relationship when studying LBW, although only change of father’s economic status was analyzed then [12].

We found that mothers with preterm history presented a fourfold increase in probability to repeat LBW. The study by Krymo et al. [11] identified an increased risk for prematurity repetition in cases where preterm history was identified. Although our study showed that intermediate or inadequate quality of antenatal care was associated with LBW recurrence, Bakewell et al. [10] evaluating whether the mother attended antenatal care and the gestational age at the onset of prenatal care did not find association with LBW recurrence. Weight gains over 10 kg during pregnancy seem to be a protective factor for LBW recurrence, in agreement with Bakewell et al. [10] findings, where the higher the weight gain in the second gestation, the lower the risk for LBW repetition.

Limitations of the study must be highlighted. First, since our conclusions rely on information from the past, like mother’s own birthweight and gestational age as well as occurrence of LBW in previous pregnancies, recall bias might have happened. On the other hand, efficient mother’s recruitment methods in the hospitals coupled with the fact that more than 99% of Pelotas deliveries take place in these hospitals, strengthened our results for the studied population.

Second, the small number of LBW infants did not allow for separate analyses that would account for the etiologically heterogeneous character of LBW (mothers with previous LBW fetuses at term and mothers with previous LBW who were born preterm may present different prognostic factors). Furthermore and for the same reason, potential etiologic differences related to the severity of LBW (infants weighing 1500–2499 g likely differ etiologically from infants weighing <1500 g) were not accounted for in the study. Furthermore, the number of previous LBW newborns experienced by women with ≥2 previous deliveries was not considered. Nonetheless, interaction between all independent variables including parity was tested and none were statistically significant.

## Conclusion

Although this study cannot answer whether the probability of LBW repetition depends on the mother’s persistence in risk factors or whether the risk for LBW recurrence would be the same if those factors could be changed in successive gestations, our results point out that appropriate antenatal care and adequate maternal weight gain in pregnancy may help to prevent recurrence of LBW among high risk mothers.

## Abbreviations

LBW: Low birth weight; SGA: Small for gestational age; PR: Prevalence ratio; 95% CI: 95% confidence interval.

## Competing interests

The authors declare that they have no competing interests.

## Authors’ contributions

IKTS conceived the study, participated in its coordination, performed the statistical analysis, and draft the first version of the manuscript. ISS participated in the design and coordination of the study and helped to draft the manuscript. MRD participated in the coordination of the study and in the statistical analysis. AM and AJDB participated in the design and coordination of the study. All authors read and approved the final manuscript.

## Pre-publication history

The pre-publication history for this paper can be accessed here:

http://www.biomedcentral.com/1471-2393/13/20/prepub
